# Are two or four hands needed for elderly female bystanders to achieve the required chest compression depth during dispatcher-assisted CPR: a randomized controlled trial

**DOI:** 10.1186/s13049-016-0238-z

**Published:** 2016-04-11

**Authors:** Asta Krikscionaitiene, Zilvinas Dambrauskas, Tracey Barron, Egle Vaitkaitiene, Dinas Vaitkaitis

**Affiliations:** Department of Disaster Medicine, Lithuanian University of Health Sciences, Eiveniu 4-512, Kaunas, LT 50161 Lithuania; Department of Surgery, Lithuanian University of Health Sciences, Kaunas, Lithuania; International Academies of Emergency Dispatch, Bristol, UK

**Keywords:** Chest compressions, CPR quality, Dispatcher-assisted CPR, Bystander, Basic life support, Manikin, Four-hands CC

## Abstract

**Background:**

Rescuers are often unable to achieve the recommended 5–6 cm CC depth. The physical limitations of elderly bystanders may affect the quality of CC; thus, we investigated new strategies to improve CC performance.

**Methods:**

We performed a randomized controlled trial in December 2013. Sixty-eight lay rescuers aged 50–75 were randomized to intervention or control pairs (males and females separately). Each pair performed 8 min of DA-CPR on a manikin connected to a PC. Each participant in every pair took turns performing CCs in cycles of 2 min and switched as advised by the dispatcher. In the middle of every 2-min cycle, the dispatcher asked the participants of the intervention group to perform the Andrew’s manoeuvre (to push on the shoulders of the person while he/she performed CCs to achieve deeper CC). Data on the quality of the CCs were analysed for each participant and pair.

**Results:**

The CC depth in the intervention group increased by 6.4 mm (*p* = 0.002) compared to the control group (54.2 vs. 47.8 mm) due to a significant difference in the female group. The CC depth in the female intervention and control groups was 51.5 and 44.9 mm.

**Discussion:**

The largest group of out-of-hospital cardiac arrest occurred in males over the age of 60 at home, and accordingly, the most likely witness, if any, is the spouse or family member, most frequently an older woman. There is a growing body of evidence that female rescuers are frequently unable to achieve sufficient CC depth compared to male rescuers. In some instances, the adequate depth of the CCs could only be reached using four hands, with the second pair of hands placed on the shoulders of the rescuer performing CPR.

**Conclusion:**

Andrew’s manoeuvre (four-hands CC) during the simulated DA-CPR significantly improved the performance of elderly female rescuers and helped them to achieve the recommended CC depth.

## Background

Bystander-initiated cardiopulmonary resuscitation (CPR) is a vital link in improving survival for victims of out-of-hospital cardiac arrest (OHCA) [1]. For every 30 people who receive bystander CPR, 1 additional life is saved [2]. Medical emergency dispatcher (MED) telephone instructions to callers increase bystander CPR rates and survival after OHCA [3]. Recent data provide strong support for the long-term mortality benefit of a dispatcher CPR instruction strategy consisting of chest compression (CC) alone rather than compression plus rescue breathing among adult patients with OHCA [4]. Where instructions are required for an adult victim, dispatchers should provide hands-only CPR (HO-CPR) instructions [5]. However, HO-CPR by itself appears to be more physically demanding than conventional 30:2 CPR [6–8]. Current guidelines recommend to push the chest to a depth of 5–6 cm [5], which is more difficult to achieve not only for young motivated medical students [9, 10] but also for health care professionals [11]. Our previous study showed that a simple 5-s Andrew’s manoeuvre (when the instructor pushed on the shoulders of the CC performing student during the training session) significantly improved the performance of the female rescuers and helped them to achieve the recommended CC depth [12]. On the basis of the results of the study, we hypothesized that deeper CCs could be reached using two rescuers, with the second pair of hands placed on the shoulders of the rescuer performing CC continuously.

The primary outcome of our study was mean CC depth. We hypothesized that the use of Andrew’s manoeuvre (four-hands CC) could be effective in achieving the recommended CC depth compared to standard HO-CPR (two-hands CC technique) during DA-CPR in elderly lay-people who were most likely to witness cardiac arrest [13].

## Methods

### Study design

We performed a randomized controlled trial comparing bystander CC quality using Andrew’s manoeuvre (four-hands CC) versus standard HO-CPR (two-hands CC) during simulated DA-CPR in a population aged 50 or older.

The Regional Ethics Committee approved the study (Protocol No. BC-MF-188/2011), and all participants provided written informed consent.

### Setting

The setting was two classes in the Department of Disaster Medicine of the Lithuanian University of Health Sciences (LUHS).

### Sample size

The sample size was calculated based on the data of our previous research [12], which identified a 6.4 mm (52.9+/−6.8 vs. 46.6+/−8.0 mm) increase in CC depth using Andrew’s manoeuvre. To detect a similar change, at an error of 5 % and a power of 80 %, we estimated that a minimal sample size of at least 20 participants in each group was required.

### Study participants. Inclusion and exclusion criteria

Lay-people aged 50 or older were recruited from August to November 2013. We posted an invitation letter with an online Google registration form and shared it using Facebook.

The inclusion criteria were: (1) male or female participants aged 50 or older, with or without previous CPR training; (2) participants must score 3 or less on the validated Clinical Frailty Scale (the least frail group of elderly) as assigned by the investigators after interviewing the participants [14]. Exclusion criteria were: (1) musculoskeletal condition precluding the ability to kneel down and perform CC, including status after joint replacement; (2) cardiovascular condition precluding the ability to exert a moderate effort, including previous MI; (3) inability to go up the stairs to the fourth floor where the study was performed; and (4) arterial blood pressure >180/110 mmHg, heart rate <50 and >120 min prior to testing.

We screened the participants for the inclusion and exclusion criteria. Next, we divided the participants in pairs based on their preferences (whom they wanted to work with in a pair) and/or arrival time (males and females separately) and assigned them to a control or intervention group in a randomized fashion. Pairs were randomized employing a randomization list created with Research Randomizer (www.randomization.com) and allocated to either control or intervention group. The pair assignments were concealed in opaque envelopes. After randomization, one member of each pair chose one of the 2 opaque envelopes with the letter A or B. Participants were aware that a pair member who received A would start CPR first during testing, but were unaware of the allocation to the intervention and control groups until the end of the test.

### Testing

**Fig. 1:**
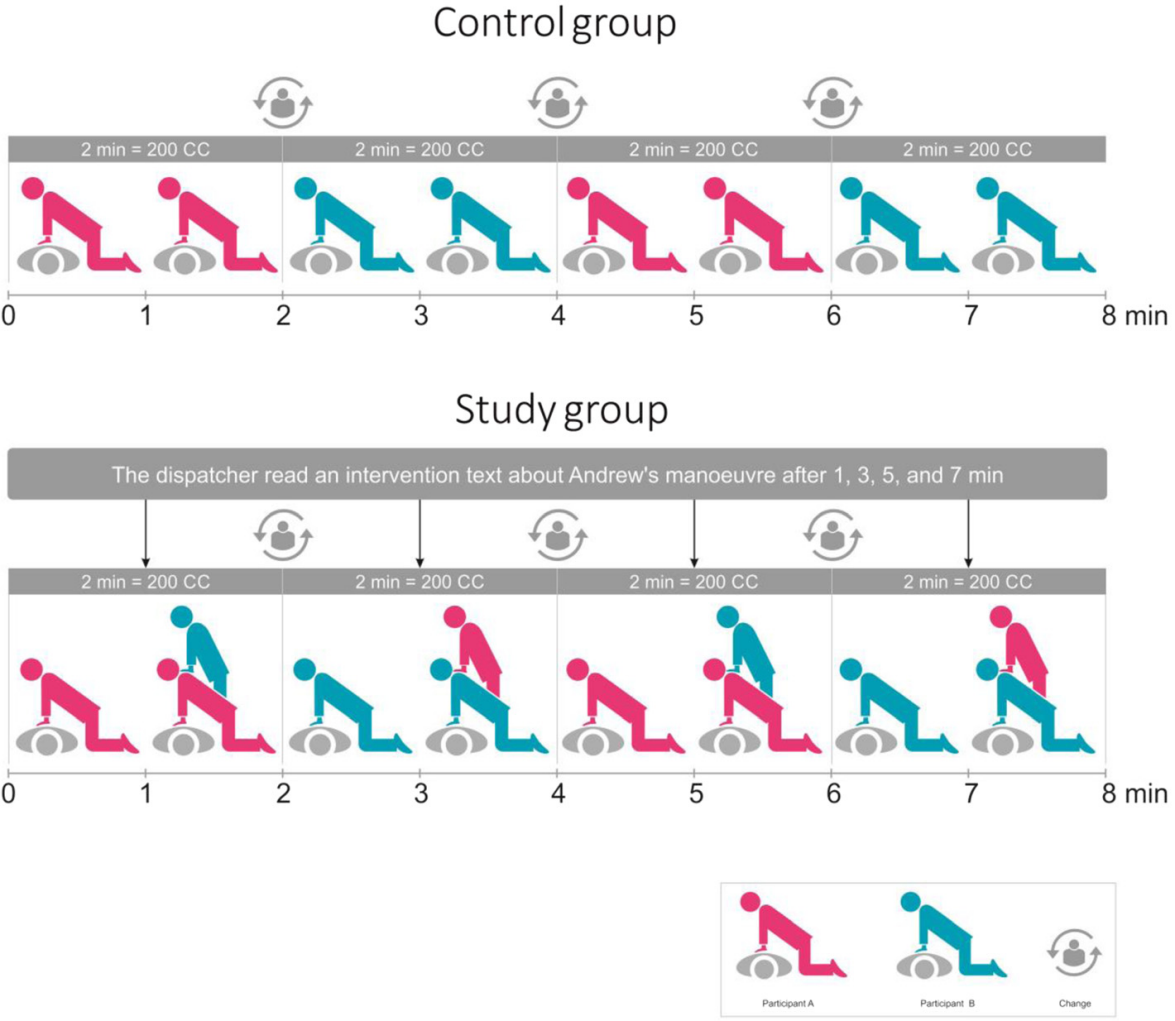
Testing flow chart

After the registration and randomization procedures, the participants were informed that they would perform DA-CPR in pairs and would be prompted to switch between each other at regular intervals. A flow chart of the testing is presented in Fig. 1. Testing was performed in two identical classrooms, with two investigators present in each room. The participants were informed that during the testing, they would only be able to communicate with the medical dispatcher over the telephone and the investigators would not answer their questions. Two certified emergency medical dispatchers from Kaunas EMS who were trained to provide the standard and four-hands CC instructions, received the phone calls from the investigators in their emergency dispatch center located 5 km away from the study setting. On the basis of the randomization results, the investigator informed the EMS dispatcher to read out the standard text (control group) or standard plus CC instructions with Andrew’s manoeuvre (intervention group). Next, the instructor placed an iPhone 4 with the loudspeaker turned on next to the head of the manikin and invited the participants into the classroom. The instructor prompted the dispatcher (by saying “Start”) to begin the simulation and the dispatcher read the standard MPDS ProQA® software (v12.1, 2010 release) text aloud: “Listen carefully, and I’ll tell you how to do resuscitation. Place the heel of your hand on the breastbone in the centre of his chest, right between the nipples. Place your other hand on top of that hand. Push down firmly 5 cm with only the heel of your lower hand touching the chest. Now, listen carefully. Pump the chest hard and fast, at least twice per second. Let the chest come up all of the way between pumps. We’re going to do this until help can take over. Count out loud so that I can count with you. One, two, three, four….”.

The dispatcher asked the participant to switch every 2 min according to MPDS ProQA® protocol. The test lasted for 8 min. (4 cycles of 2 min) and was stopped by the dispatcher. CCs were performed on a resuscitation manikin placed on the floor. Each participant had to complete two CC cycles of 2 min (4 min in total).

To the intervention group pairs, the dispatcher read an intervention text: “Is there anybody else next to the victim who could help you? OK, continue performing compressions. The other person should now stand behind you and place his hands on your shoulders. He should press on your shoulders as you perform compressions to make them deeper. Are the instructions clear? Let’s start: one two three four.” during testing in the middle of every 2-min CC cycle (approximately after 80 CC).

### Outcome measures

We measured the following baseline characteristics: age, gender, height (m), weight (kg), body mass index, and Clinical Frailty Scale (range 1–9) [14]. We also measured the heart rate (beats per minute), blood pressure (mm Hg), and saturation (%) before and 5 min after the CPR test. Each participant completed a brief survey before and after the CPR test to evaluate his own ability to perform CC, perceived stress and fatigue level (after test), dispatcher’s assistance and Andrew’s manoeuvre (only for intervention group). We used the Likert scale (range 1–5) in our survey.

CC quality data (rate, depth, leaning, hands-off time) were collected automatically using the Laerdal Resusci Anne Manikin PC SkillReporter™ System (Laerdal Medical, Stavanger, Norway) for each participant and every pair. We quantified the total CC number, CC with adequate depth, mean CC depth, CC rate, and leaning of every participant every 2 min. We also manually quantified the mean CC depth, CC rate, and leaning separately for two vs. four hands CC episodes for every participant in the intervention group for 1 min. The chest compression fraction (CCF), hands-off time, and average compression duty cycle was quantified for every pair for the entire 8-min test (although we were not able to assess the average CCF for every participant). The primary outcome was the mean CC depth. The appropriate depth was defined as 5–6 cm, according to the current guidelines [5].

### Statistical analysis

Statistical analyses were performed using SPSS 13.0 software (SPSS, Chicago, IL, USA). The data were tested for normality using the Shapiro-Wilk test. Normally distributed data were presented as the mean and standard deviation (SD); otherwise, data were presented as the median and interquartile range (IQR). To investigate associations between variables, we used t-tests and chi-squared tests. Significance was accepted at *p* < 0.05.

## Results

### Characteristics of the study subjects

**Fig. 2:**
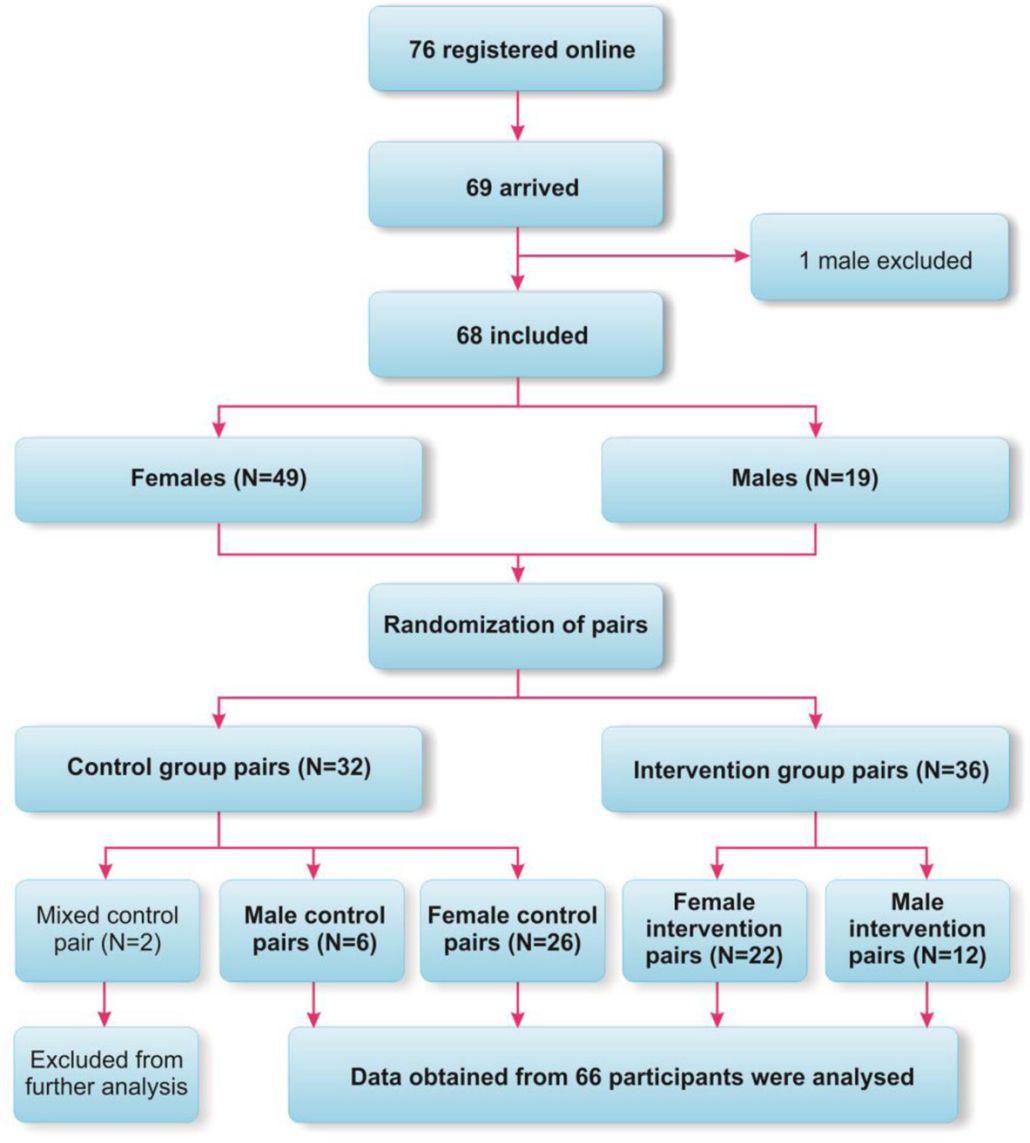
Study protocol

Sixty-eight lay-rescuers were considered for participation in the study (Fig. 2). There were 49 female and 19 male participants. One male was excluded from the study due to high blood pressure. A description of all study groups is presented in Table 1. Male and female groups were comparable according to age, height, weight, and Frailty scale. There were 4 females in the control group who had previous BLS training (median time from the last training was 24 months). Other participants had no prior BLS training experience. Data obtained from 66 bystanders was used for further analysis.

**Table 1:** Comparison of the control and intervention groups at the beginning of the study

Variable	All control group	All intervention group	*p*	Female control group	Female intervention group	*p*	Male control group	Male intervention group	*p*
	(N-32)	(N-34)		(N-26)	(N-22)		(N-6)	(N-12)	
Mean age, years	59.6(6.9)	60.6(6.9)	0.565	60.4(7)	61.7(0.2)	0.511	56.6(6.3)	58.6(7.7)	0.565
Weight, kg	79.0(16.5)	81.1(15.2)	0.562	78(17.7)	75.09(12.07)	0.519	84.8(5.1)	92.1(14.4)	0.299
Height, m	1.68(0.07)	1.69(0.1)	0.603	1.66(5.5)	1.64(5.96)	0.141	1.77(0.07)	1.79(0.08)	0.649
BMI	28.0(5.9)	28.3(4.4)	0.832	28.2(6.43)	28.0(4.67)	0.914	27.1(2.1)	28.7(4.1)	0.384
Clinical frailty score									
No 1–2 (%)	27(79.4)	24(70.6)	0.401^a^	23(85.2)	14(63.6)	0.157^a^	4(57.1)	10(83.3)	0.211^a^
No 3 (%)	7(20.6)	10(29.4)		4(14.8)	8(36.4)		3(42.9)	2(16.7)	
Heart rate, min^−1^	77.5(10.6)	79.6(11.7)	0.443	76.4(10.54)	80.2(12.10)	0.245	81.9(10.6)	78.5(11.2)	0.531
MAP, mm Hg	96.2(13.1)	91.8(11.5)	0.144	93.1(10.96)	88.4(11.00)	0.146	108.0(14.8)	97.8(10.1)	0.092
Saturation, %	97.2(1.8)	96.7(1.8)	0.636	97.3(1.88)	97.3(1.80)	0.965	96.7(1.5)	96.4(1.6)	0.697

Data are presented as the mean (SD)

^a^Chi square = 2.00, df =1

### CPR quality variables

Overall, the CCs in the intervention group were significantly deeper (54.2 vs. 47.8 mm) and CC number with adequate depth was higher (334 vs. 188) compared to the control group (Table 2). Statistical analysis revealed no differences between the intervention and control groups for the CC rate and leaning. There were no significant differences between the intervention and control pairs in CCF, hands-off time and average compression duty cycle (Table 3).

**Table 2:** Comparison of the CC quality data and vital signs after the study

Variable	All control group	All intervention group	*p*	Female control group	Female intervention group	*p*	Male control group	Male intervention group	*p*
	(N-32)	(N-34)		(N-26)	(N-22)		(N-6)	(N-12)	
Total CC number	394(56)	444(112)	0.831	390(59)	419(119)	0.001	407(44)	505(58)	0.002
CC with adequate depth, No	188(169)	334(173)	0.012	136(147)	267(157)	0.004	350(129)	501(6)	0.010
CC with adequate depth, %	46.9(41.3)	74.8(31.8)	0.003	34.9(36.8)	65.1(33.0)	0.021	84.9(31.0)	99.0(1.0)	0.218
Mean compression depth, mm	47.8(9.6)	54.2(7.1)	0.002	44.9(8.8)	51.5(7.4)	0.008	57.8(3.2)	59.3(1.2)	0.158
Mean CC rate, min^−1^	97.3(17.2)	91.0(35.6)	0.352	95.9(28.5)	93.9(28.5)	0.770	102.7(6.6)	116.8(9.2)	0.005
Leaning, %	1.1(3.6)	0.8(1.9)	0.639	1.3(4.1)	0.5(1.2)	0.372	0.4(0.4)	1.3(2.7)	0.377
Heart rate, min^−1^	80.7(12.8)	83.6(16.1)	0.427	99.1(12.3)	93.3(13.2)	0.255	87.1(13.7)	84.1(21.2)	0.738
MAP, mmHg	97.3(13.0)	93.2(12.6)	0.192	93.5(9.8)	90.9(12.8)	0.419	112.0(14.0)	97.6(11.4)	0.025
Saturation, %	97.3(1.1)	97.1(1.7)	0.866	97.0(1.1)	97.0(1.8)	0.930	97.0(0.8)	97.3(1.7)	0.716

Data are presented as the mean (SD)

**Table 3:** CC quality data of pairs

Variable	All control group pairs	All intervention group pairs	*p*	Female control group pairs	Female intervention group pairs	*p*	Male control group pairs	Male intervention group pairs	*p*
	(N-15)	(N-21)		(N-12^a^)	(N-11)		(N-3)	(N-10^a^)	
Chest compression fraction, %	85.6(7.2)	86(6.8)	0.882	84.6(7.5)	84.4(7.4)	0.956	89.5(5.3)	89.2(4.6)	0.930
Hands-off time, sec.	69.1(34.6)	67.2(32.6)	0.882	73.8(35.9)	74.8(35.5)	0.956	50.3(25.4)	52(22.2)	0.930
Average CC duty cycle, %	40.8(5.4)	44(3.3)	0.083	40.4(5.8)	44(3.6)	0.138	42.3(3.2)	44(3.4)	0.538

Data are presented as the mean (SD)

Data are presented as the median and interquartile range (IQR)

^a^There was 1 female pair from the control group and 2 male pairs from the intervention group with both members performing only every fourth CC whose results were not analyzed

The female rescuers in the intervention group, in which the Andrew’s manoeuvre was employed, achieved the recommended CC depth compared to control group (51.5 vs. 44.9 mm). The CC depth of every male in both groups exceeded 50 mm and did not differ between groups.

### Results of the survey

**Fig. 3:**
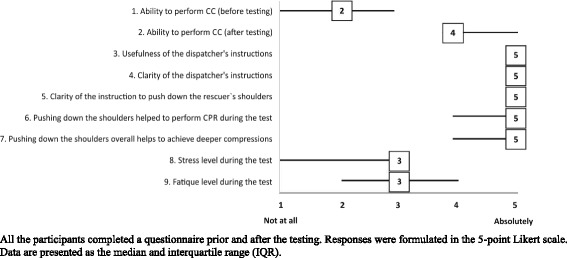
Results of the Survey. All the participants completed a questionnaire prior and after the testing. Responses were formulated in the 5-point Likert scale. Data are presented as the median and interquartile range (IQR)

All of the participants completed the anonymous questionnaire prior to and after testing; thus, we were unable to analyse the responses based on the group. The questions and answers (median value of Likert scale 1–5) of all 66 participants are presented in Fig. 3. Most of the participants had no prior CPR training and their ability to perform CC significantly improved after testing, 4.0(1.0) vs. 1.0(2.0) according to the Likert scale. The participants of the study stated that the stress level, median 3.0(2.0) and fatigue 2.5(1.0), were moderate. They highly rated the medical dispatcher’s instructions for their usefulness, 5.0(00), and clarity, 5.0(00). The participants of the intervention group described the instructions on Andrew's manoeuvre as very clear, 5.0(00), and useful during CPR, 5.0(0.75). They stated that Andrew’s manoeuvre appeared to help to achieve deeper CCs, 5.0(00).

## Discussion

The aim of the study was to evaluate the effectiveness of Andrew’s manoeuvre on CPR quality during simulated DA-CPR. We found that the CC depths of the female rescuers in the intervention group were on average 6.53 mm deeper compared to the control group, and the CCs achieved the required depth. Such an increase was significant and clinically relevant because Edelson et al. have shown that every 5-mm increase in CC depth doubled the odds of successful defibrillation [15]. Moreover, Vadeboncoeur and al has shown that each 5-mm increase in the mean CC depth significantly increased the odds of survival and survival with a favourable functional outcome [16].

The largest group of OHCA occurred in males over the age of 60 at home, and accordingly, the most likely witness, if any, is the spouse or family member, most frequently an older woman [13, 17, 18]. There is a growing body of evidence that female rescuers are frequently unable to achieve sufficient CC depth compared to male rescuers [6, 8, 19, 20]. The physical limitations of older family bystanders may affect the quality of CPR [13] and result in a worse outcome after in-home OHCA [13]. In some instances, the adequate depth of the CCs could only be reached using four hands, with the second pair of hands placed on the shoulders of the rescuer performing CPR.

Takei found that the presence of multiple rescuers was predominantly associated with good-quality bystander CPR [21]. Lerner reported that approximately half of the callers (53 %) were not alone [22]. Current CPR guidelines recommended that the provider switches approximately every 2 min [5] to limit the decrement in CPR quality over time. However, new data have shown a significant decline in CC quality during the first minute of HO-CPR [6, 7]. We support the notion that rescuers change or other intervention [23] is needed earlier than every 2 min, particularly for older laypersons or lightweight females [6, 9, 20]. The latter is the reason why we applied Andrew’s manoeuvre in the middle of each 2 min cycle. Interestingly, half of all of the intervention group pairs who started four-hands CC during the first cycle of CCs never switched back to two-hands CC. On the basis of the responses of the participants, we speculated that one of the reasons they preferred Andrew’s manoeuvre could be that it was less physically demanding to perform CPR for two persons. However, further research is needed.

Bystanders understood how to apply Andrew’s manoeuvre and performed it correctly, which suggests that Andrew’s manoeuvre could be applicable in DA-CPR. We speculated that the manoeuvre might be ideal for the normally physically incapable, and enable them to assist in CPR even if they were unable to perform it themselves.

According to the MPDS ProQA® software CPR protocol, the dispatcher asked the participants to count CCs loudly from 1 to 4 or counted by himself if the bystanders did not keep the recommended CC rate. We observed that it was difficult for the participants to count CCs aloud and to listen to the instructions of the dispatchers because they often paused when the dispatcher started speaking. Although the hands-off time in the intervention group did not statistically differ from the control group, there is a need for a more detailed investigation on the effect of the dispatcher instructions on the hands-off time. It was difficult for the participants to count to four in the Lithuanian language because the word “four” in Lithuanian has three syllables and is more difficult to pronounce. It would be easier to count to three in Lithuanian. We also observed that there were some issues related to the synchronization between the dispatcher and bystander and these issues should be investigated in detail separately.

To the best of our knowledge, this is the first reported randomized study that analyses the effectiveness of four-hands CC in the presence of multiple rescuers.

### Study limitations

The participants’ resuscitation skills were evaluated in a simulated situation; however, their abilities during actual resuscitation are unknown. The sample size of the male participants was insufficient to evaluate the effects of Andrew’s manoeuvre on males. Recruiting rescuers via the internet.

A speakerphone was used for all of the participants, which whilst necessary for this study, is not always possible during real DA-CPR. Holding a phone whilst trying to perform Andrew’s manoeuvre may not be as effective.

The test only lasted for eight minutes, and the average response time in Kaunas was 11 min.^1^ Thus, the data did not demonstrate the effectiveness of the manoeuvre over realistically longer time periods.

## Conclusions

Andrew’s manoeuvre (four-hands CC) during simulated DA-CPR significantly improved the performance of the elderly female rescuers and helped them to achieve the CC depth that is required by the current resuscitation guidelines.

## Footnotes

1 Data obtained from Kaunas EMS database.
